# Lipedema as a hormone-sensitive stromal disorder: a four-pathway translational framework

**DOI:** 10.3389/fcell.2026.1903835

**Published:** 2026-07-22

**Authors:** Diogo Pinto da Costa Viana, Adriana Luckow Invitti, Eduardo Schor

**Affiliations:** 1 Department of Gynecology, Escola Paulista de Medicina, Federal University of São Paulo (EPM-UNIFESP), São Paulo, Brazil; 2 Brazilian Society for Research and Teaching in Medicine (SOBRAPEM), São Paulo, Brazil

**Keywords:** adipose tissue, aromatase, chronic inflammation, estrogen receptor alpha, estrogen receptor beta, gynecologic–endocrine comorbidity, intracrine steroid metabolism, lipedema

## Abstract

Lipedema is a chronic disorder of subcutaneous adipose tissue characterized by disproportionate fat accumulation, pain, and progressive functional impairment, predominantly affecting women. Research remains fragmented across vascular, hormonal, metabolic, and gynecologic perspectives. Recent contributions have advanced specific axes: an international Delphi consensus, a systematic review of hormonal hypotheses, a stromal-vulnerability narrative, and a focused review of adipose biology; but no prior framework has integrated these domains into a single architecture annotated by level of evidence and capable of generating stratified, falsifiable research hypotheses. Here, we propose a hypothesis-generating translational framework that conceptualizes lipedema as the predominant adipose expression of a hormone-sensitive stromal vulnerability. The framework adds a specific molecular convergence axis, ERα/ERβ signaling imbalance interacting with intracrine steroid metabolism (aromatase, 17β-HSDs, AKR1C1), and resolves the disorder into four interacting biological pathways: (i) hormonal transition sensitivity across the female life course; (ii) metabolic–behavioral amplification; (iii) gynecologic–endocrine comorbidity; and (iv) intrinsic stromal–adipose susceptibility. The framework predicts that lipedema and its cognate expressions in hormone-responsive tissues (including gynecologic disease, connective tissue laxity, microvascular dysfunction, neurosensory amplification, and neuropsychological burden) may share a common stromal-endocrine substrate while preserving phenotypic specificity through dominant-pathway combinations. Four features distinguish this framework from prior syntheses: (a) ERα/ERβ signaling imbalance is articulated as a candidate molecular convergence axis linking adipose, gynecologic, connective-tissue, microvascular, and neuro-immune manifestations; (b) intracrine steroid metabolism (aromatase, 17β-HSDs, AKR1C1) is incorporated as the mechanistic anchor of stromal hormone-responsiveness; (c) per-component evidence-level annotation is applied throughout (Level 1A/1B: direct evidence in lipedema; Level 2: observational association; Level 3: mechanistic extrapolation); and (d) stratified, falsifiable research hypotheses are derived from dominant-pathway phenotypes. Two domains, metabolic burden and steroid signaling, emerge as promising translational research domains. Important limitations apply. Direct mechanistic evidence in lipedema-specific tissues is limited; much of the supporting biology is extrapolated from adipose, gynecologic, and metabolic literature; and most clinical data derive from observational cohorts in referral centers. This article therefore proposes a hypothesis-generating translational framework, not a clinical guideline or therapeutic recommendation.

## 1 Introduction

Lipedema is a chronic disorder of the subcutaneous adipose tissue (SAT) characterized by symmetrical and disproportionate fat accumulation, predominantly affecting the lower extremities of women and frequently accompanied by pain, edema, and increased tissue sensitivity (Allen and Hines, 1940; Wold et al., 1951; Buck and Herbst, 2016). It remains underdiagnosed and is often misclassified as obesity, lymphedema, or chronic venous disease (Katzer et al., 2021; Faerber et al., 2024; Kruppa et al., 2026).

Histological and molecular evidence consistently demonstrates that lipedematous adipose tissue exhibits distinct biological features differentiating it from both normal adiposity and obesity, including adipocyte hypertrophy, extracellular matrix (ECM) remodeling, chronic low-grade inflammation, and microvascular alterations (Szél et al., 2014; AL-Ghadban et al., 2019; Felmerer et al., 2020; Strohmeier et al., 2022). Growing evidence indicates that these alterations extend beyond adipocytes to the stromal microenvironment as a whole, with reproducible features of connective tissue laxity, ECM dysregulation, and stromal–mesenchymal vulnerability across independent cohorts (Rabiee, 2025; Fiengo and Sbarbati, 2026).

The disease occurs almost exclusively in women and frequently emerges or worsens during reproductive transitions, implicating sex steroid signaling in disease expression (Patton et al., 2024; Lüchinger et al., 2026). Within lipedematous tissue, alterations in the ERα/ERβ signaling imbalance and increased intracrine estradiol production mediated by aromatase and 17β-hydroxysteroid dehydrogenases have been described (Katzer et al., 2021; Vieira-Potter et al., 2026). At the genetic level, the candidate gene AKR1C1, coding for an aldo-keto reductase that inactivates progesterone and dihydrotestosterone within adipose tissue, has been associated with lipedema susceptibility (Kaftalli et al., 2023).

Beyond endocrine alterations within adipose tissue, clinical observations increasingly suggest that lipedema frequently coexists with gynecologic and endocrine disturbances. Observational cohorts report increased prevalence of menstrual irregularities, dysmenorrhea, polycystic ovary syndrome (PCOS), uterine fibroids, and thyroid dysfunction (Patton et al., 2024; Viana et al., 2026b). In the largest cohort described to date (n = 1803), connective tissue laxity (95.8%), thyroid disorders, and inflammatory ovarian dysfunction were reported with markedly elevated frequencies (Simarro Blasco et al., 2025).

A substantial proportion of affected individuals also present with metabolic burden (overweight, obesity, insulin resistance, and reduced mobility) which may amplify inflammatory and fibrotic signaling within lipedematous depots (Nono Nankam et al., 2022; Viana et al., 2025b; 2026a). Despite this complexity, current research remains fragmented across vascular, hormonal, metabolic, and clinical domains, with limited integration into a coherent conceptual structure (Kruppa et al., 2026).

Recent syntheses have advanced parts of this field, each along a specific axis. Kruppa et al. (2026) operationalize international agreement on definition and management through a multinational Delphi consensus. Lüchinger et al. (2026) provide a rigorous systematic synthesis of hormonal hypotheses across the lipedema literature. Fiengo and Sbarbati (2026) advance the perspective that lipedema may represent the predominant adipose expression of a broader hormone-sensitive stromal vulnerability involving connective, vascular, endocrine, and neuropsychological domains. Rabiee (2025) offers a concise review of current adipose-biology controversies and future directions.

The present framework explicitly builds on the systemic-stromal perspective articulated by Fiengo and Sbarbati (2026) and integrates it with the gynecologic–endocrine synthesis proposed in the scoping review by Viana et al. (2026b) and the conceptual model proposed by Viana et al. (2025a), which articulated menopause as a critical turning point through ERα/ERβ signaling imbalance, intracrine estrogen, and adipose tissue dysfunction. To this conceptual ground, it adds four structural features that, to the best of our knowledge, have not been combined in a single prior synthesis: (i) ERα/ERβ signaling imbalance is articulated as a candidate molecular convergence axis linking adipose, gynecologic, connective-tissue, microvascular, and neuro-immune manifestations within a hormone-sensitive stromal microenvironment; (ii) intracrine steroid metabolism (aromatase, 17β-HSDs, AKR1C1) is incorporated as the mechanistic anchor of stromal hormone-responsiveness, providing a molecular substrate for the systemic vulnerability; (iii) per-component evidence-level annotation (Level 1A/1B; Level 2 and Level 3; see Table 2) is applied throughout the framework; and (iv) stratified, falsifiable research hypotheses are derived from dominant-pathway phenotypes. The framework is positioned as complementary to the recent contributions cited above rather than as their re-litigation.

To address this gap, we propose a translational four-pathway framework anchored on a working hypothesis: that a hormone-sensitive stromal vulnerability, molecularly convergent at the ERα/ERβ signaling imbalance and mechanistically anchored on intracrine steroid metabolism, manifests preferentially in subcutaneous adipose tissue as lipedema, while expressing in parallel as cognate manifestations in other hormone-responsive tissues. Four pathways modulate magnitude, timing, and combination of these expressions: (1) hormonal transitions across the female life course; (2) metabolic–behavioral amplification; (3) gynecologic–endocrine comorbidity; and (4) intrinsic stromal–adipose susceptibility. The article proposes a hypothesis-generating framework, not a clinical guideline or therapeutic recommendation.

### 1.1 Source selection and methodological transparency

This synthesis was developed iteratively from a structured search of PubMed, Embase, Web of Science, Scopus, and Cochrane Library (January 2014 – February 2026), complemented by foundational historical references (Allen and Hines, 1940; Wold et al., 1951; O’Brien et al., 1998). Search terms combined lipedema-specific descriptors with hormonal, metabolic, gynecologic, stromal, and steroid-metabolism keywords. Studies were prioritized when they provided (a) lipedema-specific mechanistic data (Level 1), (b) observational lipedema data (Level 2), or (c) mechanistic findings from adipose, gynecologic, or metabolic literature relevant to the framework but not directly validated in lipedema (Level 3). Cross-citations within the recent lipedema literature (Rabiee, 2025; Fiengo and Sbarbati, 2026; Kruppa et al., 2026; Lüchinger et al., 2026; Viana et al., 2026b) were used to identify additional sources.

Per Frontiers Hypothesis and Theory format, no formal systematic-review methodology was applied; however, the explicit per-component level-of-evidence annotation (Tables 1–3) makes transparent the inferential weight of each component. The authors have previously contributed to this field (Viana et al., 2025a; 2025b; 2026a; 2026b); a potential confirmation bias inherent to this position is acknowledged and mitigated through (i) independent dual review in the underlying scoping review, (ii) transparent labeling of self-citations, and (iii) explicit evidence-level annotation throughout the present manuscript.

**TABLE 1:** Translational synthesis of the four biological pathways contributing to lipedema expression.

Pathway	Biological mechanisms	Supporting evidence	Translational implications
Stromal microenvironment vulnerability (with adipose-predominant expression)	Adipocyte hypertrophy; ECM remodeling and interstitial fibrosis; chronic low-grade inflammation; microvascular and endothelial barrier dysfunction; ERα/ERβ signaling imbalance as candidate molecular convergence axis; intracrine estradiol production; polygenic stromal susceptibility (e.g., AKR1C1); cognate expressions in connective, vascular, and neuro-immune tissues	Szél et al. (2014), AL-Ghadban et al. (2019), Felmerer et al. (2020), Katzer et al. (2021), Chakraborty et al. (2022), Strohmeier et al. (2022), Kaftalli et al. (2023), Michelini et al. (2025), Fiengo and Sbarbati (2026), Vieira-Potter et al. (2026)	Identification of unifying biological substrate; mechanistic targets in ERα/ERβ signaling imbalance, ECM remodeling, and intracrine steroid metabolism
Hormonal transition sensitivity	Disease onset/progression associated with puberty, pregnancy, and menopause; altered systemic and intracrine estrogen signaling	Patton et al. (2024), Rabiee (2025), Lüchinger et al. (2026), Vieira-Potter et al. (2026)	Investigation of life-course endocrine modulation in lipedema biology
Metabolic–behavioral amplification	Insulin resistance; chronic low-grade inflammation; metabolic rigidity of adipose tissue; mitochondrial inflexibility; mobility limitation due to pain	Keuper et al. (2017), Nawaz et al. (2017), Cornely et al. (2022), Poojari et al. (2022), Chachaj et al. (2024), Viana et al. (2025b)	Exploration of metabolic interventions targeting inflammatory–metabolic pathways
Gynecologic–endocrine comorbidity	Menstrual disturbances; pelvic pain clusters; PCOS; uterine fibroids; impaired progesterone responsiveness; dysregulated steroid metabolism	O’Brien et al. (1998), Patton et al. (2024), Simarro Blasco et al. (2025), Viana et al. (2026b)	Investigation of endocrine phenotypes and steroid-modulating strategies in selected subgroups

**TABLE 2:** Evidence-level framework for components labeled in Figure 1.

Level	Definition	Components labeled in Figure 1	Representative studies
Level 1A	Clinical description or historical anchor study in lipedema	Initial phenotypic delimitation of disproportionate lower-limb adiposity	Allen and Hines (1940), Wold et al. (1951)
Level 1B	Direct mechanistic, molecular, histological, or imaging evidence in lipedema-specific tissues	ECM remodeling and fibrosis; intracrine estradiol; ERα/ERβ signaling imbalance; AKR1C1 and steroid metabolism; interstitial sodium expansion; endothelial dysfunction; adipocyte hypertrophy	Szél et al. (2014), AL-Ghadban et al. (2019), Crescenzi et al. (2020), Felmerer et al. (2020), Katzer et al. (2021), Wolf et al. (2021), Strohmeier et al. (2022), Kaftalli et al. (2023)
Level 2	Observational association in lipedema (cohort, cross-sectional, registry, or consensus data without mechanistic validation)	Life-transition associations; gynecologic–endocrine comorbidity clusters (PCOS, fibroids, thyroid dysfunction); connective-tissue laxity; bariatric-resistant adiposity; functional impairment and reduced mobility	Pouwels et al. (2018), Cornely et al. (2022), Poojari et al. (2022), Chachaj et al. (2024), Patton et al. (2024), Rabiee (2025), Simarro Blasco et al. (2025), Fiengo and Sbarbati (2026), Lüchinger et al. (2026), Viana et al. (2026b)
Level 3	Mechanistic extrapolation from adipose, gynecologic, or metabolic literature, biologically plausible but not yet validated in lipedema	PR-A/PR-B substrate in SAT; intracrine estradiol pathway; M2 macrophage signaling; GLP-1/GIP adipose remodeling; mineralocorticoid receptor effects; progesterone-resistance biology; steroid-modulating mechanisms	O’Brien et al. (1998), Bulun et al. (2006), Brown et al. (2012), Brown and Farquhar (2014), Infante et al. (2017), Keuper et al. (2017), Makabe et al. (2017), Nawaz et al. (2017), Drucker (2018), Samms et al. (2020), Rosenstock et al. (2021), Chung and Han (2022), Jastreboff et al. (2022), Parasiliti-Caprino et al. (2022), Viana et al. (2025b), Viana et al. (2026a)
Cross-cutting	Practice guidelines and Delphi consensus informing definition and management of lipedema	Diagnostic criteria; clinical staging; standardized management framework	Faerber et al. (2024), Kruppa et al. (2026)

The table defines each level, maps it to the corresponding components of the four-pathway model, and lists representative studies.

**TABLE 3:** Stratified research hypotheses derived from the four-pathway framework.

Clinical phenotype	Dominant pathway	Key biological mechanisms	Research hypothesis (illustrative class)	Level
Early-stage lipedema with strong hormonal triggers	Hormonal transition sensitivity	ERα/ERβ signaling imbalance; intracrine estradiol production; reproductive endocrine fluctuations	Test whether steroid-signaling modulation alters disease trajectory at puberty/pregnancy/perimenopause	Level 3
Lipedema with prominent menstrual disturbances and pelvic symptoms	Gynecologic–endocrine comorbidity	Impaired progesterone responsiveness; altered steroid metabolism; overlap with hormone-sensitive gynecologic disorders	Test whether steroid-modulating agents (progestins; agents acting on aromatase/17β-HSD) influence adipose-stromal endpoints	Level 3
Lipedema associated with obesity or metabolic syndrome	Metabolic–behavioral amplification	Insulin resistance; chronic inflammation; metabolic rigidity of adipose tissue	Test whether metabolic-targeted therapies affect tissue stiffness, pain, and limb composition	Level 3
Lipedema with systemic metabolic dysregulation and inflammatory burden	Metabolic–behavioral amplification	Chronic inflammation; mitochondrial dysfunction; impaired adipose remodeling	Test whether dual incretin receptor agonism (GLP-1/GIP class) modifies adipose-tissue inflammation and remodeling	Level 3
Advanced lipedema with reduced mobility, dynapenic phenotype	Metabolic–behavioral amplification (advanced)	Inflammatory myosteatosis; adipose-muscle crosstalk; mitochondrial inflexibility	Test whether anti-inflammatory or metabolic-reconditioning strategies modify muscle quality and functional capacity	Level 3
Lipedema with combined endocrine and metabolic disturbances	Interaction of metabolic and gynecologic pathways	Crosstalk between steroid signaling, inflammation, and metabolic dysregulation	Test whether multimodal strategies combining metabolic and steroid-modulating components affect stratified endpoints	Level 3

The table illustrates how distinct dominant pathways generate distinct hypothesis-driven research questions, not clinical recommendations.

A terminological clarification is warranted. The term “lipedema” — retained throughout this manuscript for consistency with international diagnostic nomenclature (ICD-11 EF02.2; ICD-10 E88.20–E88.22) and global indexing—has been increasingly characterized as a misnomer, since true interstitial oedema is variable, inconsistent across patients, and not pathognomonic of the condition. Alternative designations have been proposed, most notably “lipohyperplasia dolorosa” (LiDo), which better reflects the genetically determined, hyperplastic, and painful nature of the disease (Cornely et al., 2022; Brenner et al., 2023). We acknowledge this active terminological debate and the validity of the LiDo framing. We retain “lipedema” here primarily for indexing and continuity with the cited primary literature, while explicitly noting that the present framework concerns the broader hormone-sensitive stromal vulnerability proposed to underlie lipedema/LiDo, not the interstitial-oedema component per se.

## 2 Conceptual framework of lipedema biology

**FIGURE 1:**
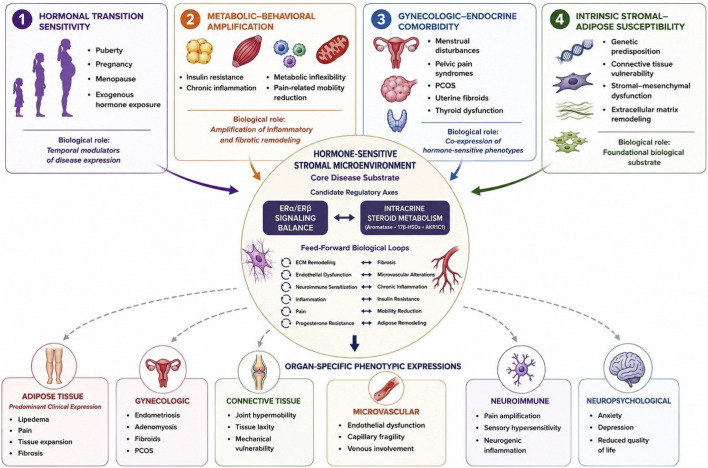
Lipedema as the predominant adipose expression of a hormone-sensitive stromal vulnerability: a four-pathway convergence model with ERα/ERβ signaling imbalance as a candidate molecular convergence axis.

Lipedema cannot be sufficiently explained by a single biological mechanism or by an exclusively adipose-centered model. The framework interprets lipedema as the predominant adipose expression of a hormone-sensitive vulnerability of the stromal microenvironment (Fiengo and Sbarbati, 2026), arising from the interaction between an intrinsic stromal–adipose susceptibility, molecularly convergent at the ERα/ERβ signaling imbalance and anchored on intracrine steroid metabolism, and systemic modulators acting across the female life course. Four interacting biological domains (stromal–adipose vulnerability, hormonal transitions, metabolic amplification, and gynecologic–endocrine comorbidity) converge on a progressive inflammatory and fibrotic microenvironment that gives rise to heterogeneous clinical phenotypes. This architecture is depicted in Figure 1, which organizes these domains around the hormone-sensitive stromal core and maps their organ-specific cognate expressions.

### 2.1 Stromal–adipose vulnerability

Lipedematous adipose tissue exhibits adipocyte hypertrophy and hyperplasia, ECM remodeling, microvascular alterations, and chronic inflammatory infiltration (Szél et al., 2014; Felmerer et al., 2020; Nono Nankam et al., 2022). These features extend beyond the adipocyte to involve the stromal–vascular fraction, consistent with a broader hormone-sensitive stromal vulnerability preferentially expressed in adipose tissue but not confined to it (Rabiee, 2025; Fiengo and Sbarbati, 2026). Within this microenvironment, dysregulated steroid signaling has been documented, including altered ERα/ERβ signaling imbalance and increased intracrine estradiol production via aromatase and 17β-hydroxysteroid dehydrogenases (Katzer et al., 2021; Vieira-Potter et al., 2026), alongside expression of progesterone receptor isoforms PR-A and PR-B in subcutaneous adipose tissue (O’Brien et al., 1998), providing a direct substrate for stromal hormone responsiveness. Stromal–adipose vulnerability therefore represents the biological substrate on which additional systemic modulators act, occupying the central position as the core of the disease.

For operational purposes within this framework, the hormone-sensitive stromal vulnerability is defined as a coordinated alteration of the stromal–vascular fraction encompassing: (i) ECM remodeling and progressive interstitial fibrosis; (ii) altered ERα/ERβ signaling and dysregulated intracrine steroid metabolism; (iii) endothelial barrier dysfunction with increased capillary permeability and interstitial fluid accumulation; (iv) chronic low-grade immune cell infiltration with elevated chemotactic cytokines (e.g., MCP-1, TNFSF14, EN-RAGE); and (v) neurogenic inflammation with mast cell activation and small-fiber involvement. These components are not proposed as a unitary lesion but as a coordinated pattern of stromal–endocrine dysregulation that may be expressed in varying combinations and intensities across patients and tissues. The boundaries of this construct are addressed in Section 4.1.

### 2.2 Systemic modulators of disease expression

Three systemic domains interact with the stromal–adipose vulnerability. First, hormonal transition sensitivity: lipedema onset and progression frequently coincide with puberty, pregnancy, and menopause (Patton et al., 2024; Lüchinger et al., 2026). Second, metabolic–behavioral amplification: overweight, insulin resistance, and reduced mobility may amplify inflammatory and fibrotic signaling within affected depots (Nono Nankam et al., 2022; Viana et al., 2025b). Third, gynecologic–endocrine comorbidity: observational studies report increased frequencies of menstrual disturbances, pelvic pain, PCOS, uterine fibroids, and thyroid dysfunction in women with lipedema (Patton et al., 2024; Simarro Blasco et al., 2025; Viana et al., 2026b).

These domains operate as interacting components of a dynamic network rather than independent causal pathways: in some individuals, hormonal transitions predominate; in others, metabolic amplification or gynecologic–endocrine clustering. Table 1 summarizes the four domains, their mechanisms, supporting evidence, and translational implications.

Rather than proposing a single mechanistic explanation, the framework organizes current evidence to facilitate hypothesis generation, mechanistic investigation, and stratified clinical research.

## 3 Biological pathways underlying lipedema expression

Figure 1 presents the mechanistic four-pathway architecture centered on the hormone-sensitive stromal microenvironment, with ERα/ERβ signaling imbalance proposed as one of the leading candidate molecular convergence axes. It is structured around three elements: (i) the central core (hormone-sensitive stromal microenvironment), comprising ERα/ERβ signaling imbalance, intracrine steroid metabolism, and the interconnected processes of ECM remodeling, endothelial dysfunction, and neuroimmune sensitization; (ii) the four interacting biological domains converging on this core; and (iii) the organ-specific cognate expressions of the shared stromal-endocrine substrate: adipose tissue (lipedema) as the predominant site and gynecologic, connective-tissue, microvascular, neuroimmune, and neuropsychological manifestations as parallel organ-specific expressions. The following subsections develop each pathway, with components labeled Level 1, Level 2, or Level 3 per the criteria defined in Section 3.5 and Table 2.

The central hormone-sensitive stromal microenvironment (core of disease) is organized around ERα/ERβ signaling imbalance and intracrine steroid metabolism (aromatase, 17β-HSDs, AKR1C1), within which ECM remodeling and fibrosis, endothelial dysfunction, and neuroimmune sensitization operate as interconnected processes. Four interacting biological domains converge upon this core: (1) hormonal transition sensitivity, (2) metabolic–behavioral amplification, (3) gynecologic–endocrine comorbidity, and (4) intrinsic stromal susceptibility. Pathway components are detailed in the corresponding panels. Model output: organ-specific cognate tissue expressions of the shared stromal-endocrine substrate, with adipose tissue (lipedema) as the predominant site of clinical manifestation; cognate expressions in gynecologic, connective tissue, microvascular, neuroimmune, and neuropsychological domains are shown in the lower panels. Core principle: a systemic hormone-sensitive stromal vulnerability in which ERα/ERβ signaling imbalance represents one candidate convergence mechanism that, together with intracrine steroid metabolism, may underlie multiple organ-specific manifestations, with adipose tissue being the predominant site of clinical expression in lipedema. Per-component evidence levels (Level 1A/1B: direct evidence in lipedema; Level 2: observational association; Level 3: mechanistic extrapolation) follow criteria defined in Section 3.5 and Table 2. Figure 1 is intentionally schematic; arrows represent biologically plausible relationships proposed within the hormone-sensitive stromal substrate, not experimentally demonstrated causal mechanisms (see Section 6, Limitations, point 7). Abbreviations: SAT, subcutaneous adipose tissue; ERα/ERβ, estrogen receptor alpha/beta; ECM, extracellular matrix; HSD, hydroxysteroid dehydrogenase; PCOS, polycystic ovary syndrome.

### 3.1 The hormone-sensitive stromal microenvironment as a unifying substrate

Lipedema cannot be fully accounted for by adipocyte biology alone. Histological and microvascular studies show adipocyte hypertrophy and hyperplasia, ECM remodeling, interstitial fibrosis, microvascular alterations, endothelial barrier dysfunction, and stromal–mesenchymal vulnerability (Szél et al., 2014; AL-Ghadban et al., 2019; Felmerer et al., 2020; Wolf et al., 2021; Strohmeier et al., 2022; Michelini et al., 2025; Nono Nankam et al., 2022). Building on Fiengo and Sbarbati (2026), we interpret these alterations as a hormone-sensitive vulnerability of the stromal microenvironment, preferentially expressed in subcutaneous adipose tissue but not confined to it.

Within this interpretation, the stromal microenvironment (fibroblasts, adipose-derived stromal cells, pericytes, endothelial cells, immune cells, and ECM) functions as a unifying biological substrate whose dysregulated hormone-responsiveness shapes disease expression across multiple tissues. Lipedema represents the predominant adipose expression; gynecologic disease, connective-tissue laxity, microvascular dysfunction, and neurosensory amplification may represent organ-specific cognate expressions (Section 3.1.2). This does not negate the adipose–vascular paradigm instrumental for clinical recognition (Faerber et al., 2024; Kruppa et al., 2026), but situates it within a broader interpretive structure.

Mechanistically, this vulnerability is hormone-responsive. Subcutaneous adipose tissue expresses progesterone receptor isoforms (O’Brien et al., 1998) (PR-A and PR-B), aromatase (CYP19A1) and 17β-hydroxysteroid dehydrogenases mediating intracrine estradiol biosynthesis (Vieira-Potter et al., 2026), and the steroid-inactivating enzyme AKR1C1 (Kaftalli et al., 2023). These elements provide a molecular substrate through which local steroid signaling can be amplified or attenuated independently of circulating hormone levels. Polygenic susceptibility is suggested by genome-wide and candidate-gene studies (Kaftalli et al., 2023; Fiengo and Sbarbati, 2026), supporting a multifactorial architecture.

#### 3.1.1 ERα/ERβ signaling imbalance as a candidate molecular convergence axis

Within the stromal microenvironment, ERα/ERβ signaling imbalance emerges as a plausible molecular convergence axis requiring further validation in lipedema-specific tissues, alongside complementary contributions from intracrine steroid metabolism, progesterone-receptor function, and mineralocorticoid-receptor signaling. The two isoforms exert partially opposing effects: ERα predominantly mediates protective metabolic activity, adipogenic differentiation, and vascular integrity, while ERβ favors pro-fibrotic, pro-angiogenic, and pro-inflammatory programs (Vieira-Potter et al., 2026). A relative ERβ predominance has been described in lipedematous tissue (Katzer et al., 2021; Rabiee, 2025) and parallel ERβ-dominant signaling is documented in estrogen-dependent gynecologic disorders such as endometriosis and adenomyosis (Bulun et al., 2006; Chung and Han, 2022). A focused mechanistic model articulating this imbalance in the menopausal context has been proposed (Viana et al., 2025a) and is integrated here within the broader four-pathway architecture.

If confirmed experimentally, ERα/ERβ signaling imbalance may modulate, within a single regulatory axis, several processes relevant to lipedema and its cognate manifestations, including ECM remodeling, microvascular permeability and endothelial integrity (Strohmeier et al., 2022; Michelini et al., 2025), angiogenesis and lymphangiogenesis (AL-Ghadban et al., 2019; Chung and Han, 2022), connective-tissue mechanotransduction, neuroimmune signaling and sensory sensitization (Chakraborty et al., 2022; Dinnendahl et al., 2024), and adipogenesis and lipid storage (Vieira-Potter et al., 2026). Local intracrine estradiol biosynthesis via aromatase and 17β-HSDs may further contribute to sustaining ERβ-dominant signaling independently of circulating hormone levels, potentially reconciling pronounced tissue-level effects with apparently normal systemic estradiol concentrations.

Direct demonstration of ERα/ERβ signaling imbalance in lipedema tissue derives primarily from focused mechanistic reviews and limited histological evidence (Katzer et al., 2021; Rabiee, 2025); a substantial portion of the supporting biology is extrapolated from gynecologic and adipose literature and is classified Level 3. The proposal therefore advances ERα/ERβ as one specific testable hypothesis among several candidate endocrine regulators that require experimental validation—including mineralocorticoid-receptor signaling (Infante et al., 2017; Parasiliti-Caprino et al., 2022) and progesterone-receptor function (O’Brien et al., 1998) — and as a structured framework to guide biomarker-driven research, not as an established mechanism or master regulator. Specific candidate biomarkers, expected findings, and falsifying findings associated with this and other components of the framework are summarized in Table 4.

**TABLE 4:** Candidate biomarkers and falsifiable predictions across the four pathways of the framework.

Pathway/Component	Candidate biomarker	Tissue/Sample	Expected finding	Falsifying finding	Evidence level/References
ERα/ERβ axis (Pathway 4, core)	ERα and ERβ mRNA and protein expression ratio	Lipedematous SAT biopsy + BMI-matched control SAT	Lower ERα/ERβ ratio in lipedematous tissue vs. controls	Identical or higher ERα/ERβ ratio vs. controls	L1B/Katzer et al., 2021; Rabiee, 2025
Intracrine steroid metabolism (Pathway 4, core)	Aromatase (CYP19A1), 17β-HSD1, 17β-HSD2, AKR1C1 mRNA/protein	Lipedematous SAT biopsy	Increased aromatase + 17β-HSD1; reduced 17β-HSD2; AKR1C1 variants more frequent	No enzymatic dysregulation vs. controls	L2/L3/Kaftalli et al., 2023; Vieira-Potter et al., 2026; Viana et al., 2025a
Local intracrine estradiol (Pathway 4, core)	Tissue estradiol (LC-MS/MS) and tissue/serum estradiol ratio	SAT homogenate + matched serum	Elevated intra-tissue estradiol with normal or low circulating estradiol (dissociation)	No dissociation; tissue levels track serum levels	L3/Vieira-Potter et al., 2026
Stromal inflammatory–metabolic dissociation (Pathways 2 + 4)	TNFSF14, CASP8, EN-RAGE, MCP-1, VEGFA, TGFβ1 vs. HbA1c, adiponectin	Serum + matched anthropometry	Elevated inflammatory/angiogenic markers despite preserved metabolic profile (replicated)	No inflammatory elevation, or full coupling with HbA1c rise	L1B/Nono Nankam et al., 2022
ECM remodeling and fibrosis (Pathway 4, core process)	Type I/III collagen ratio, MMP-2/9, TIMP-1, hyaluronic acid	SAT biopsy histology + serum	Increased fibrillar collagen and altered MMP/TIMP balance	No remodeling signature; collagen architecture preserved	L1B/Felmerer et al., 2020; Strohmeier et al., 2022
Microvascular dysfunction (Pathway 4, core process)	Capillary density, VEGFA, ICAM-1, VCAM-1; endothelial barrier integrity	SAT histology + serum + imaging	Increased capillary density and permeability; elevated endothelial markers	No microvascular alteration vs. controls	L1B/AL-Ghadban et al., 2019; Strohmeier et al., 2022; Michelini et al., 2025
Neurogenic inflammation (Cognate; neuroimmune)	Mast cell density (tryptase IHC), CGRP, substance P; QST profile	SAT biopsy + sensory testing (non-obese stage I–II)	Increased mast cell density; altered QST profile in non-obese patients	No neurogenic involvement; normal QST profile	L1B/Chakraborty et al., 2022; Dinnendahl et al., 2024
Interstitial fluid and tissue sodium (Cognate; vascular)	Tissue sodium MRI quantification (^23^Na-MRI)	*In vivo* MRI of affected limbs	Elevated tissue sodium correlating with stage and pain severity	No elevation; no correlation with stage or pain	L1B/Crescenzi et al., 2020
GIPR signaling (Pathway 2, therapeutic relevance)	GIPR mRNA/protein expression in adipocytes	Lipedematous SAT biopsy	Reduced or impaired GIPR expression vs. BMI-matched controls	Normal GIPR expression (would weaken metabolic-pathway predictions for tirzepatide)	L3/El et al., 2023 (mechanism precedent)
Connective-tissue cognate expression (Cognate; Pathway 4)	Beighton score ≥5 (joint hypermobility)	Clinical cohort vs. age-matched general female population	Higher prevalence in lipedema (Beighton ≥5; OR ≥3 vs. general population ∼10–20%)	Prevalence similar to general female population	L2/Aday et al., 2024 (OR 12.88); Patton et al., 2024; Simarro Blasco et al., 2025
Pain-amplification cognate expression (Cognate; neuroimmune)	ACR criteria fibromyalgia prevalence	Clinical cohort vs. age-matched general female population	Prevalence ∼30–40% (substantially higher than general population ∼2–5%)	Prevalence comparable to general population (∼2–5%)	L2/Cagliyan Turk et al., 2024 (35%)

Each row identifies a measurable biomarker, its tissue/sample type, the finding expected under the proposed framework, the finding that would constitute evidence against it, the current evidence level (L1B: direct evidence in lipedema-specific tissues; L2: observational association in lipedema; L3: mechanistic extrapolation), and the supporting literature. These predictions are intentionally formulated to allow direct experimental adjudication.

#### 3.1.2 Cognate expressions across hormone-responsive tissues

If a hormone-sensitive stromal vulnerability constitutes a shared substrate, the framework predicts that lipedema should frequently co-occur with cognate manifestations in other hormone-responsive tissues. Recent observational and mechanistic evidence is compatible with this prediction, though it remains insufficient to establish causal directionality and should be interpreted with caution.

Importantly, co-occurrence of clinical manifestations alone cannot be interpreted as evidence of a shared biological substrate. The proposed stromal-endocrine convergence remains a testable hypothesis requiring molecular validation across tissues.

Connective tissue laxity and hypermobility are reported with high frequency across independent lipedema cohorts: 95.8% in the Spanish cohort of Simarro Blasco et al. (2025); in a cohort of 1803 patient, 31.59% Beighton ≥ 5 in the Italian cohort of Patton et al. (2024), and a markedly increased odds ratio of joint hypermobility (OR 12.88) in women with lipedema compared with controls (Aday et al., 2024). Fiengo and Sbarbati (2026) documented hypermobile body regions in 44% of patients and historical childhood hypermobility in approximately 60%, suggesting that connective-tissue features may be underestimated in adulthood owing to progressive fibrosis. Whether these features correspond to defined connective-tissue disorders such as hypermobile Ehlers–Danlos syndrome remains uncertain and has not been systematically established (Fiengo and Sbarbati, 2026).

Microvascular and endothelial alterations have been documented histologically (AL-Ghadban et al., 2019; Strohmeier et al., 2022; Michelini et al., 2025), and chronic venous disease is reported in 71.9%–86.2% of patients (Patton et al., 2024; Fiengo and Sbarbati, 2026). Neurosensory amplification, including neuropathic pain features in 43.3% (Erden et al., 2025), neurogenic inflammation (Chakraborty et al., 2022), and altered quantitative sensory testing profiles in non-obese patients (Dinnendahl et al., 2024), supports involvement of small-fiber and stromal-neural interactions consistent with a sensitized stromal microenvironment. Fibromyalgia, which shares features of hormone-sensitive pain amplification and stromal dysregulation, has been diagnosed in approximately 35% of patients with lipedema (Cagliyan Turk et al., 2024). Gynecologic comorbidity is detailed in Section 3.4 (Patton et al., 2024; Simarro Blasco et al., 2025; Viana et al., 2026b). Gastrointestinal and neuropsychological manifestations have also been reported across multiple cohorts (Patton et al., 2024; Fiengo and Sbarbati, 2026).

Alternative systemic hypotheses have been proposed within the same conceptual neighborhood, including the endotoxin–complement cascade model of Kruglikov and Scherer (2024), which proposes that circulating endotoxins and complement activation may contribute to lipedematous adipose expansion. Such models are not mutually exclusive with the present framework: within a shared stromal-endocrine substrate, immunometabolic and neuro-immune mechanisms may contribute in a context-dependent manner.

Several alternative explanations must be considered for each cognate manifestation discussed above and should not be dismissed. Connective-tissue features may reflect independent genetic predisposition unrelated to lipedema biology, including overlapping but distinct heritable connective-tissue disorders. Gastrointestinal and neuropsychological symptoms may have independent etiologies, including chronic-pain-related mechanisms, medication effects, and selection bias toward more symptomatic patients in specialized referral centers. Fibromyalgia overlap may reflect shared chronic-pain pathways and central sensitization rather than a unifying stromal-endocrine substrate. The framework does not claim to explain these manifestations causally; it predicts that they should cluster with measurable stromal-endocrine signatures in dominant-pathway phenotypes and that their prevalences should exceed those reported for age-matched general female populations (joint hypermobility ∼10–20%; fibromyalgia ∼2–5%) — a quantitative prediction directly testable in observational and biomarker-driven studies that would also distinguish the framework from these alternative explanations.

It must be emphasized that the cognate expressions outlined here represent observed associations and biologically plausible parallels, not established causal links. Their inclusion is meant to articulate the predictive scope of the framework and to identify research priorities, not to assert clinical equivalence between lipedema and these conditions. The framework predicts that patients with prominent connective-tissue features should preferentially express dominant Pathway 4 (intrinsic stromal–adipose susceptibility), and that patients with prominent gynecologic comorbidity should preferentially express dominant Pathway 3, a prediction that biomarker-stratified studies could test directly.

### 3.2 Hormonal transition sensitivity

Lipedema frequently emerges or worsens during puberty, pregnancy, and the menopausal transition (Patton et al., 2024; Lüchinger et al., 2026) (Figure 1, pathway 1). In an international Delphi consensus, experts identified life-course hormonal influences as a central domain in lipedema biology (Kruppa et al., 2026). Within the present framework, these transitions are interpreted not as primary causes but as biological revealing events that expose a pre-existing stromal–adipose susceptibility.

During puberty, rapid increases in circulating estradiol drive gluteofemoral adipose expansion. In predisposed individuals, this physiological process may become dysregulated, with disproportionate and persistent lower-limb fat deposition and clinical features such as easy bruising emerging in adolescence (Katzer et al., 2021). Pregnancy adds substantial estrogen and progesterone load alongside systemic insulin resistance and adipose expansion; symptom exacerbation during gestation is frequently reported (Patton et al., 2024).

Menopause has received increasing attention as a potential turning point. The transition combines declining ovarian estradiol with shifts in receptor signaling, redistribution of adipose toward central depots, and chronic low-grade inflammation (Vieira-Potter et al., 2026). In lipedematous tissue, this may shift the receptor balance further toward ERβ-mediated inflammatory and fibrotic programs while local intracrine estradiol production sustains tissue-level signaling (Katzer et al., 2021; Rabiee, 2025; Viana et al., 2025a). Symptom fluctuations have also been reported with exogenous hormones, although findings are heterogeneous and likely depend on formulation, dose, and individual hormonal sensitivity (Patton et al., 2024; Lüchinger et al., 2026).

Hormonal transition sensitivity therefore acts as the temporal regulator of disease expression: endocrine milestones unmask or amplify pathological processes within a susceptible stromal–adipose microenvironment.

### 3.3 Metabolic–behavioral amplification

Despite intrinsic resistance to caloric restriction and exercise—which often fail to reduce lipedematous fat compartments even after substantial bariatric weight loss (Pouwels et al., 2018; Cornely et al., 2022) — a substantial proportion of patients present with concurrent obesity, insulin resistance, and chronic low-grade inflammation (Patton et al., 2024) (Figure 1, pathway 2). Importantly, recent evidence indicates that lipedema cannot be reduced to a metabolic phenotype of obesity. Nono Nankam et al. (2022) demonstrated that women with lipedema, compared with age- and BMI-matched controls, exhibit a paradoxical biological signature: preserved or improved glycemic regulation (lower HbA1c, higher adiponectin) coexisting with significantly elevated circulating inflammatory proteins (including TNFSF14, CASP8, EN-RAGE, MCP-1, IL-8, TGFα, TGFβ1, and VEGFA) and oxidative stress markers (malondialdehyde, superoxide dismutase, catalase). This dissociation between metabolic and inflammatory–oxidative profiles is consistent with the present framework, in which the predominant driver is hormone-sensitive stromal inflammation and tissue remodeling rather than metabolic obesity per se. It also supports the interpretation that lipedema and coincident obesity, when present, represent two distinct biological conditions in the same individual rather than a continuum, with BMI-based assessment recently challenged in favor of waist-to-height-ratio-based criteria (Brenner et al., 2023). Excess adiposity nonetheless amplifies disease expression: it promotes macrophage activation, cytokine release, and ECM remodeling in adipose tissue (Keuper et al., 2017; Nawaz et al., 2017), processes that overlap with the inflammatory microenvironment of lipedema (Szél et al., 2014; Felmerer et al., 2020; Wolf et al., 2021).

Behavioral factors interact with these processes. Chronic pain reduces physical activity and increases disability in women with lipedema relative to BMI-matched controls (Chachaj et al., 2024), exacerbating insulin resistance and inflammation in self-reinforcing cycles (feed-forward dynamics linking inflammation, insulin resistance, and pain-driven mobility reduction) that may culminate, in advanced stages, in inflammatory myosteatosis and a dynapenic phenotype (Viana et al., 2026a). At the cellular level, lipedematous adipocytes may exhibit reduced oxidative capacity and impaired thermogenic signaling, consistent with the clinical observation of diet-resistant fat (Poojari et al., 2022; Viana et al., 2025b).

Metabolic amplification thus functions as a secondary but clinically relevant driver: it does not initiate disease but accelerates inflammatory and fibrotic remodeling within the susceptible stromal–adipose microenvironment.

### 3.4 Gynecologic–endocrine comorbidity

Observational cohorts have reported a substantial burden of gynecologic symptoms and diagnoses in women with lipedema (menstrual disturbances, dysmenorrhea, PCOS, uterine fibroids, thyroid dysfunction, and infertility) at frequencies exceeding general-population estimates (Patton et al., 2024; Viana et al., 2026b) (Figure 1, pathway 3). In the largest available cohort (n = 1803), connective tissue laxity (95.8%), chronic low-grade inflammatory alterations, and inflammatory ovarian dysfunction were reported, alongside elevated rates of thyroid disorders and psychological impairment (Simarro Blasco et al., 2025).

Mechanistic parallels with hormone-sensitive gynecologic disorders are noteworthy. Endometriosis and adenomyosis share altered ER signaling, progesterone resistance, increased aromatase expression, and reduced 17β-HSD2 activity, sustaining local estradiol bioavailability (Bulun et al., 2006; Chung and Han, 2022). The presence of PR-A and PR-B in human subcutaneous adipose tissue (O'Brien et al., 1998), together with AKR1C1-related inactivation of progesterone in adipose tissue (Kaftalli et al., 2023), provides a plausible molecular substrate for impaired progesterone modulation within lipedematous depots, operationalized as a bidirectional dynamic between progesterone-resistance biology and adipose-stromal remodeling. These parallels suggest shared endocrine vulnerability rather than disease equivalence (Viana et al., 2026b).

Gynecologic–endocrine comorbidity therefore functions as a co-expression domain rather than a downstream consequence: it points to a shared molecular substrate between lipedema and concomitant hormone-sensitive gynecologic disease, and supports integrated assessment across both domains.

### 3.5 Hierarchy of evidence supporting the framework

To make explicit the inferential weight of each claim, supporting studies are classified at four evidence levels and applied throughout Figure 1 as Level 1, Level 2, and Level 3 badges. Level 1A refers to clinical descriptions or historical anchor studies in lipedema cohorts. Level 1B refers to direct mechanistic, molecular, histological, or imaging evidence in lipedema-specific tissues. Level 2 refers to observational association in lipedema (cohort, cross-sectional, registry, or consensus data without mechanistic validation). Level 3 refers to mechanistic extrapolation from adipose, gynecologic, or metabolic literature in which findings are biologically plausible but have not been directly validated in lipedema. A cross-cutting category groups practice guidelines and Delphi consensus informing definition and management. Table 2 defines each level, maps it to the corresponding components labeled in Figure 1.

## 4 Interactions between biological pathways

Although presented separately, the four domains are best understood as a dynamic interaction within the hormone-sensitive stromal substrate. The stromal microenvironment is the central node: ECM remodeling, inflammatory activation, microvascular dysfunction, and ERα/ERβ-mediated steroid signaling create a substrate sensitive to systemic endocrine and metabolic perturbations (Szél et al., 2014; Felmerer et al., 2020; Katzer et al., 2021; Michelini et al., 2025; Fiengo and Sbarbati, 2026). Within this core, three feed-forward dynamics operate concurrently (ECM remodeling and fibrosis, endothelial dysfunction and microvascular alterations, and neuroimmune sensitization and inflammation), all modulated by ERα/ERβ signaling imbalance and intracrine steroid metabolism. These dynamics make the bidirectional nature of the model explicit, accommodating self-reinforcing loops such as inflammation–insulin resistance, ERβ signaling–fibrosis, pain–mobility impairment, and progesterone resistance–adipose remodeling.

Hormonal transitions act as temporal modulators that shift this substrate toward inflammatory and fibrotic programs (Patton et al., 2024; Lüchinger et al., 2026). Metabolic disturbances reinforce immune signaling and ECM remodeling through immunometabolic mechanisms (Keuper et al., 2017; Nawaz et al., 2017). Gynecologic comorbidities share steroid-metabolism and receptor-signaling pathways with the lipedematous niche (O’Brien et al., 1998; Kaftalli et al., 2023), suggesting that some patients may express a hormone-sensitive stromal phenotype that manifests across reproductive and adipose tissues.

These mechanisms form feed-forward loops: adipose inflammation worsens metabolic dysfunction, amplifying immune signaling and ECM remodeling; dysregulated steroid metabolism shapes both adipose biology and systemic metabolic regulation. Over time, convergence of these processes underlies progressive fibrosis, impaired lipid mobilization, and (in advanced disease) inflammatory myosteatosis and dynapenia (Viana et al., 2026a). Recognizing these interactions is essential for interpreting phenotypic heterogeneity and designing studies that account for variable domain contributions.

### 4.1 Boundary conditions and counterexamples

A robust framework must accommodate observations that resist its primary explanatory structure. Four boundary conditions deserve explicit consideration. First, although lipedema affects predominantly women, sporadic male cases have been reported; within the present framework, this is compatible with the substrate role of adipose-derived intracrine estrogen production, which provides estrogenic signaling in male adipose tissue as well (Vieira-Potter et al., 2026), and predicts that male phenotypes would express dominantly the intrinsic adipose vulnerability pathway rather than the gynecologic–endocrine pathway.

Second, a subset of women with lipedema presents without prominent gynecologic comorbidity. The framework predicts that these patients should express dominantly pathways 4 (intrinsic stromal–adipose susceptibility) and 2 (metabolic–behavioral amplification), and that their disease trajectory may be less responsive to steroid-modulating strategies and more responsive to metabolic-targeted interventions. Third, lean women with advanced lipedema challenge the centrality of the metabolic pathway; the framework accommodates these cases by predicting predominance of pathways 1 (hormonal transition sensitivity) and 3 (gynecologic–endocrine comorbidity), with reduced contribution of metabolic amplification, a configuration that should be testable through biomarker stratification.

Fourth, the well-documented persistence of lipedema-distribution adiposity and pain after substantial bariatric weight loss (Pouwels et al., 2018; Cornely et al., 2022) is, within the framework, not anomalous but predicted: bariatric surgery addresses systemic metabolic overload but does not modify the local stromal-endocrine substrate. The framework thus generates the falsifiable prediction that bariatric outcomes should correlate with pre-operative dominant-pathway phenotype, with poorer lipedema-specific outcomes in patients with predominant intrinsic adipose vulnerability or hormonal-sensitivity profiles.

These boundary conditions transform the framework from descriptive to predictive: each counterexample, rather than refuting the model, generates a specific testable hypothesis about which pathway combination is dominant and which interventions are biologically rational in that subgroup.

### 4.2 Competing interpretations of lipedema biology

A theory acquires explanatory value only against the alternatives it must outperform or accommodate. Several interpretive models of lipedema biology coexist in the current literature, each anchoring causality in a different compartment. An adipocyte-first model treats the disorder as a primary disease of adipose tissue, in which adipocyte hypertrophy, hyperplasia, and diet-resistant expansion are the initiating events and stromal, vascular, and inflammatory changes are downstream consequences (Szél et al., 2014; Felmerer et al., 2020). A vascular- and microvascular-first model emphasizes endothelial dysfunction, capillary fragility, increased permeability, and angiogenesis as the leading edge of disease, with adipose expansion and fibrosis following from chronic microvascular injury (AL-Ghadban et al., 2019; Strohmeier et al., 2022; Michelini et al., 2025). A lymphatic-first interpretation, historically influential and still embedded in clinical staging and conservative management, frames lipedema as a disorder of lymphatic load and drainage in which adipose and connective-tissue changes are secondary to subclinical lymphatic insufficiency (Allen and Hines, 1940; Forner-Cordero et al., 2012; Faerber et al., 2024). More recently, an immunometabolic endotoxin–complement model has proposed that circulating endotoxins and complement activation drive lipedematous adipose expansion through innate-immune mechanisms (Kruglikov and Scherer, 2024).

The present framework does not seek to displace these models but to provide the substrate within which each operates. The adipose, vascular, and lymphatic compartments are not competing primary sites but co-resident elements of the same hormone-sensitive stromal microenvironment, and the innate-immune mechanisms invoked by the endotoxin–complement model are compatible with the neuroimmune sensitization already represented within the central core (Section 3.1). Where the prior models specify a leading compartment, the convergence framework specifies a shared regulatory logic (ERα/ERβ signaling and intracrine steroid metabolism acting on a vulnerable stroma) that predicts why no single compartment fully accounts for the phenotype and why the dominant compartment may differ across patients. The present framework should therefore be interpreted as complementary to, rather than mutually exclusive with, the adipocyte-first, vascular-first, lymphatic-first, and endotoxin–complement interpretations; its distinguishing claim is integrative rather than competitive, and the discriminating test is whether a shared stromal-endocrine signature can be demonstrated across the compartments that the prior models treat as primary. Candidate biomarkers permitting differential weighting of these complementary models—including markers specific to lymphatic dysfunction, endothelial barrier integrity, immune activation, and steroid signaling—are summarized in Table 4.

## 5 Clinical and translational implications

The framework carries implications at two levels: how lipedema is clinically evaluated and which mechanism-driven hypotheses merit dedicated investigation. The following subsections first consider clinical evaluation, then develop two translational research domains (metabolic modulation and steroid-modulating approaches, each operating in feed-forward dynamics with the central hormone-sensitive stromal core), and finally derive stratified, falsifiable research hypotheses organized by dominant pathway (Section 5.4; Table 3).

### 5.1 Clinical evaluation

The framework supports multidimensional patient evaluation. Morphological features remain essential for diagnosis (Faerber et al., 2024; Kruppa et al., 2026), but endocrine, metabolic, and functional assessment add information on disease expression and progression. Awareness of life-course endocrine milestones may help recognize periods of symptom emergence or acceleration (Patton et al., 2024; Lüchinger et al., 2026). Metabolic parameters, body composition, and functional capacity inform disease trajectory (Cornely et al., 2022; Chachaj et al., 2024); integrated gynecologic and endocrine evaluation may identify comorbidities interacting with stromal–adipose biology (Patton et al., 2024; Simarro Blasco et al., 2025; Viana et al., 2026b).

Within this framework, heterogeneity of clinical presentation should be interpreted not as diagnostic inconsistency but as a reflection of variable contribution of interacting biological domains, supporting future efforts at phenotypic stratification.

Importantly, biological plausibility does not imply clinical efficacy. The pathways below formulate testable research hypotheses, not clinical interventions. No agent discussed has demonstrated disease-modifying activity in lipedema in randomized trials; inclusion is illustrative of mechanism-driven research questions, not clinical recommendations.

### 5.2 Metabolic modulation as a research domain

The metabolic–behavioral amplification pathway points to insulin resistance, low-grade inflammation, and mitochondrial inefficiency as candidate targets operating in feed-forward dynamics with the central stromal core. Dual GLP-1/GIP receptor agonism improves insulin sensitivity, systemic inflammatory markers, and adipose remodeling in metabolic disease (Drucker, 2018; Samms et al., 2020; Rosenstock et al., 2021; Jastreboff et al., 2022); a focused synthesis of mechanistic and translational evidence linking these pathways to lipedema and to tirzepatide’s potential relevance has been proposed (Viana et al., 2025b). Whether such effects translate to lipedema-specific outcomes (tissue stiffness, pain, limb composition) remains an L3 hypothesis requiring dedicated investigation.

An additional methodological consideration concerns the molecular determinants of incretin response in lipedematous tissue. Tirzepatide acts through dual GLP-1/GIP receptor agonism, and GIPR-mediated signaling in target tissues contributes substantially to its therapeutic effects (El et al., 2023). To our knowledge, however, GIPR expression and functional integrity in lipedematous adipocytes have not been directly characterized in published literature, which constitutes a notable evidence gap given the recognized refractoriness of lipedematous fat depots to caloric restriction and the dissociation between adipose mass reduction and lipedema-specific pain reported after bariatric weight loss (Cornely et al., 2022). Direct characterization of incretin-receptor expression in lipedematous adipose tissue is therefore included as a candidate biomarker and research priority in Table 4 — not as a presumed alteration, but as an empirically tractable question whose answer would clarify whether receptor-level mechanisms contribute to this clinical phenotype.

### 5.3 Steroid-modulating approaches as a research domain

The gynecologic–endocrine pathway positions steroid signaling as a complementary research domain in selected subgroups, operating in feed-forward dynamics with the central stromal core through ERα/ERβ signaling imbalance and intracrine steroid metabolism. In estrogen-dependent gynecologic disease, progestin-based and steroid-modulating agents modulate inflammatory and proliferative programs associated with dysregulated steroid metabolism (Brown et al., 2012; Brown and Farquhar, 2014). Drospirenone has attracted attention for its antimineralocorticoid activity, given that mineralocorticoid receptor activation contributes to adipocyte oxidative stress and pro-inflammatory adipokine secretion (Infante et al., 2017; Parasiliti-Caprino et al., 2022), and for its experimental effects on inflammatory cytokines, VEGF, and NGF in endometriotic stromal cells (Makabe et al., 2017). Other steroid-modulating molecules acting on aromatase or 17β-HSD isoenzymes are mentioned as additional examples of a broader class of agents informing mechanistic hypotheses, not as specific clinical proposals. Evidence supporting any of these molecules in lipedema is limited and derives from mechanistic parallels rather than randomized lipedema trials. Their discussion is therefore intended to inform hypothesis generation and future translational research rather than to support clinical use in lipedema at the present time.

### 5.4 Stratified research hypotheses

Within the four-pathway framework, distinct dominant pathways generate distinct research questions. Individuals with prominent metabolic amplification might benefit from biomarker-driven trials of metabolic reconditioning strategies; those with prominent endocrine comorbidity might be the target of trials testing steroid-modulating interventions. Synergistic strategies combining metabolic and endocrine modulation are biologically plausible and could inform multimodal trial designs. The model also supports the development of biomarker panels capable of stratifying patients by dominant pathway. The corresponding stratified research hypotheses are summarized in Table 3.

Accordingly, the framework should be judged not by its capacity to accommodate existing observations, but by the accuracy of its prospective molecular and biomarker-based predictions.

The framework is formulated to be falsifiable, and its central claims generate specific predictions whose failure would refute rather than merely qualify the model. The convergence hypothesis would be substantively refuted by any of the following observations in adequately powered, biomarker-driven cohorts: (1) consistent absence of reproducible ERα/ERβ imbalance in lipedematous tissue relative to matched non-lipedematous adipose tissue; (2) absence of altered intracrine steroid metabolism (aromatase, 17β-HSDs, or AKR1C1 activity) within affected depots; (3) absence of the predicted gynecologic–endocrine clustering when lipedema cohorts are compared with appropriately matched controls free of referral-center selection bias; and (4) absence of shared stromal-endocrine molecular signatures across adipose and cognate hormone-responsive tissues (gynecologic, connective, microvascular, neuroimmune) in the same individuals. A further discriminating prediction is that dominant-pathway phenotype should track measurable biology: failure of pre-specified dominant-pathway strata to differ in steroid-metabolism enzymes, inflammatory markers, or imaging-derived tissue composition would undermine the stratification logic on which the framework rests. Conversely, demonstration of these signatures (and their concentration within the subgroups predicted to express dominant pathways 1, 3, or 4) would provide direct support and would distinguish the convergence model from the compartment-specific interpretations discussed in Section 4.2. Specific candidate biomarkers, expected findings, falsifying findings, and current evidence levels are summarized in Table 4.

## 6 Limitations

Several limitations apply. First, the framework derives from synthesis of observational, mechanistic, and translational evidence rather than direct experimental validation in large lipedema cohorts. Although alterations in inflammation, steroid signaling, and metabolism have been described in lipedematous tissue (Szél et al., 2014; Felmerer et al., 2020; Katzer et al., 2021), the causal hierarchy between these mechanisms and disease progression remains undefined. Direct demonstration of ERα/ERβ imbalance in human lipedematous tissue derives primarily from focused mechanistic reviews and limited histological samples (Katzer et al., 2021; Rabiee, 2025); large-cohort validation across adipose and cognate tissues has not yet been performed.

Second, much clinical evidence on metabolic and gynecologic comorbidities comes from specialized referral centers, with selection bias toward more advanced or comorbid phenotypes (Patton et al., 2024; Viana et al., 2026b); reported frequencies may not generalize to early-stage or community populations.

Third, validated molecular or imaging biomarkers for lipedema are lacking; classifications rely on physical examination and symptom history, producing heterogeneity across studies (Faerber et al., 2024; Kruppa et al., 2026). Development of biomarkers characterizing adipose inflammation, fibrosis, and endocrine signaling is a critical next step.

Fourth, the therapeutic concepts discussed, incretin-based metabolic agents and steroid-modulating molecules, are hypothesis-generating constructs based on mechanistic extrapolation from other diseases; no agent has demonstrated disease-modifying activity in lipedema in randomized trials.

Fifth, the authors have previously published conceptual work on the hormonal and metabolic hypotheses of lipedema (Viana et al., 2025a; 2025b; 2026b; 2026a), and a potential confirmation bias inherent to that position should be acknowledged. Although several of the concepts incorporated into the present framework are supported by emerging evidence, further independent validation across diverse cohorts and research groups will be important to establish their reproducibility and generalizability. Mitigation strategies adopted in the underlying scoping review, including independent dual review, transparent use of self-citation, and methodological adherence to JBI and PRISMA-ScR guidance (Viana et al., 2026b), are extended here through explicit hierarchy-of-evidence labeling, source-selection disclosure (Section 1.1), and the inclusion of boundary conditions and counterexamples (Section 4.1).

Sixth, the cognate expressions in Section 3.1.2 (connective-tissue laxity, fibromyalgia, microvascular dysfunction, neurosensory amplification) are biologically plausible parallels supported by observational and mechanistic evidence, not established causal links. Their inclusion articulates the predictive scope of the framework and identifies directions for biomarker-driven research, not clinical equivalence between lipedema and these conditions. The relationship of lipedema to defined connective-tissue disorders such as hypermobile Ehlers–Danlos syndrome has not been systematically established (Fiengo and Sbarbati, 2026).

Seventh, Figure 1 is intentionally schematic and represents biologically plausible relationships rather than experimentally demonstrated causal mechanisms. Arrows in the diagram should be interpreted as proposed conceptual connections within the hormone-sensitive stromal substrate, not as direct causal pathways. The evidence level (Level 1A/1B, 2, or 3) for each component is detailed in Section 3.5 and Table 2. Alternative mechanistic models—including lymphatic-first, vascular-first, and endotoxin–complement interpretations (Section 4.2) — should be considered as complementary rather than mutually exclusive with the hormone-sensitive stromal hypothesis; their relative contributions remain experimentally unresolved.

Taken together, the model should be interpreted as a structured research hypothesis requiring validation through biomarker-driven cohorts, tissue-based mechanistic studies, and prospective stratified clinical research, not as an established pathophysiological model or a basis for therapeutic decisions.

## 7 Conclusion and future directions

Lipedema challenges traditional medical paradigms. The framework does not propose that lipedema is primarily a hormonal disease, a vascular disease, or a metabolic disease; instead, it proposes that these domains converge upon a shared hormone-sensitive stromal substrate whose predominant clinical expression occurs in adipose tissue. Accumulating evidence is compatible with its conceptualization as the predominant adipose expression of a hormone-sensitive stromal vulnerability. The framework integrates this systemic-stromal perspective (Fiengo and Sbarbati, 2026) with a specific molecular substrate, ERα/ERβ signaling imbalance as a candidate convergence axis anchored on intracrine steroid metabolism, and resolves the disorder into four interacting pathways (Figure 1): intrinsic stromal–adipose susceptibility, hormonal transition sensitivity, metabolic–behavioral amplification, and gynecologic–endocrine comorbidity.

By organizing these domains within a single architecture annotated by evidence level, the framework offers a structured interpretation of why lipedema co-occurs with cognate manifestations in other hormone-responsive tissues while preserving phenotypic specificity through dominant-pathway combinations. Two domains, metabolic burden and steroid signaling, emerge as potentially modifiable and define candidate axes for stratified research. The framework is offered as a hypothesis-generating structure, not a clinical guideline. Comparative analyses of stromal-endocrine biology across lipedema and concomitant hormone-sensitive gynecologic disorders may be particularly informative (Viana et al., 2026b).

Several priorities emerge. First, clarifying the molecular crosstalk between estrogen-receptor signaling and intracrine steroid metabolism in lipedematous tissue (including aromatase, 17β-HSDs, and AKR1C1) is a central research frontier (Katzer et al., 2021; Kaftalli et al., 2023; Vieira-Potter et al., 2026). Second, characterizing the stromal–mesenchymal phenotype beyond the adipocyte (ECM organization, endothelial barrier integrity, connective-tissue laxity) may sharpen the biological boundaries of the disease (Strohmeier et al., 2022; Fiengo and Sbarbati, 2026). Third, prospective cohorts with standardized endocrine and gynecologic assessment are needed to test whether patterns observed in referral centers generalize to community populations (Patton et al., 2024; Simarro Blasco et al., 2025). Fourth, biomarker-driven phenotyping integrating steroid-metabolism enzymes, inflammatory markers, mitochondrial bioenergetics, and imaging may enable stratified research. Finally, translational trials within biologically defined subgroups are needed to evaluate disease-modifying candidates, per Table 3.

## Data Availability

No original datasets were generated or analyzed in this study. All information supporting this hypothesis and theoretical framework is contained within the article. Further inquiries can be directed to the corresponding author.
